# Ethics of task shifting in the health workforce: exploring the role of community health workers in HIV service delivery in low- and middle-income countries

**DOI:** 10.1186/s12910-018-0312-3

**Published:** 2018-07-04

**Authors:** Hayley Mundeva, Jeremy Snyder, David Paul Ngilangwa, Angela Kaida

**Affiliations:** 1 Simon Fraser University, 8888 University Drive, Burnaby, BC V5A 1S6 Canada; 2 Amref Health Africa Tanzania, Ali Hassan Mwinyi Road, Plot 1019, P.O. Box 2773, Dar es Salaam, Tanzania

**Keywords:** Community health workers, HIV, Ethics, Principlism, Global health, Task shifting, Task sharing

## Abstract

**Background:**

Task shifting is increasingly used to address human resource shortages impacting HIV service delivery in low- and middle-income countries. By shifting basic tasks from higher- to lower-trained cadres, such as Community Health Workers (CHWs), task shifting can reduce overhead costs, improve community outreach, and provide efficient scale-up of essential treatments like antiretroviral therapies. Although there is rich evidence outlining positive outcomes that CHWs bring into HIV programs, important questions remain over their place in service delivery. These challenges often reflect concerns over whether CHWs can mitigate HIV through a means that does not overlook the ethical and practical constraints that undergird their work. Ethical and practical guidance thus needs to become the cornerstone of CHW deployment. This paper analyzes such challenges through the lens of Ethical Principlism.

**Methods:**

We examined papers identifying substantive and ethical challenges impacting CHWs as they provide HIV services in low- and middle-income contexts. To do this, we analyzed papers written in English and published from year 2000 or later. These articles were identified using MEDLINE, Cochrane Database of Systematic Reviews, and Google Scholar databases. In total, 465 articles were identified, 78 of which met our inclusion criteria. Article reference lists and grey literature were also examined.

**Results:**

CHWs experience specific challenges while carrying out their duties, such as conducting emotionally- and physically-demanding tasks with often inadequate training, supervision and compensation. CHWs have also been poorly integrated into health systems, which not only impacts quality of care, but can hinder their prospects for promotion and lead to CHW disempowerment. As we argue, these challenges can be addressed if a set of ethical principles is prioritized, which specifically entail the principles of respect for persons, justice, beneficence, proportionality and cultural humility.

**Conclusions:**

CHWs play a crucial role in HIV service delivery, yet the ethical challenges that can accompany their work cannot be overlooked. By prioritizing ethical principles, policymakers and program implementers can better ensure that CHWs are combatting HIV through a means that does not exploit or take their critical role within service delivery for granted.

## Background

According to the World Health Organization (WHO), 57 countries are facing severe health worker shortages, over half of which are in Africa [1]. These shortages are exacerbated by several factors including challenging working conditions, a high prevalence of communicable diseases such as HIV, or an exodus of health workers [1–3]. Many countries with fragile health systems are undertaking distinctive measures to address these challenges in hopes of improving health service provision. As reflected in the HIV epidemic, large-scale task shifting has taken place across sub-Saharan Africa in which basic HIV prevention, treatment and care responsibilities are delegated from higher- to lower-trained health workers [4, 5]. Shifting these tasks to less specialized health cadres can reduce the workloads of overburdened workers, streamline patient services [6] and ultimately improve workforce capacity within health systems [1]. Although it can take many different forms, task shifting is most notably known for causing a resurgence of community health workers (CHWs) across sub-Saharan Africa and South Asia [7], and this change has been accompanied with a unique set of triumphs as well as challenges.

A CHW is a community member who receives basic training in disease prevention, treatment and care to carry out some type of health service; however, because of their basic training, he/she is often not formally qualified as a health care professional [8]. Depending on the focus of their training, CHWs can undertake various roles. Some of these include mobilizing immunization programs [9, 10], to promoting healthy behaviour [7], to being specialized communicators for high-prevalence infections like HIV [2, 11, 12]. CHWs have been especially crucial to HIV treatment, as they have been able to fill gaps in health services during the rapid scale up in access to combination antiretroviral therapy (cART) [13].

In the context of HIV service provision, CHWs often act as essential intermediaries between community members and the formal health care delivery sector [14]. By offering a range of services from HIV testing to counseling to referrals [2, 14], CHWs can increase community knowledge about the virus and how it is transmitted, which has played a crucial role in mitigating HIV-related stigma [12]. By having strong ties to communities, CHWs have also been vital in improving cultural appropriateness of health interventions [15]. By providing further services such as adherence counseling and contact tracing, CHWs have helped to improve uptake and retention rates within HIV service provision [6]. As many scholars have further noted, efficient scale-up of antiretroviral therapies (ARTs) would simply not be feasible in resource-limited health systems if CHWs were not quickly trained, as their deployment is highly cost-effective [5, 16]. CHWs therefore represent an integral part of HIV service delivery, as they can dramatically improve access and coverage to essential services [5].

Although CHWs can efficiently address gaps in HIV service provision, their widespread involvement is accompanied with a set of challenges [2, 16, 17]. These challenges are foremost grounded in concerns over justice and fairness regarding the benefits and burdens they can encounter from their work. For example, concerns over exploitation emerge, as CHWs risk being exposed to unfair employment practices including inadequate training and unfair compensation [12]. Not only have CHWs been expected to conduct their work on a voluntary basis, but many CHWs have had their responsibilities poorly explained to them, causing some to assume roles that otherwise belong to higher paid and trained staff [18]. Such issues can lead to CHW disempowerment [19], cause unnecessary power imbalances to be perpetuated between them and other cadres [20], and ultimately result in poorer quality services being provided to clients [21].

CHWs have also been undervalued within service provision, as they have been poorly integrated into many formal health systems [6, 11, 12, 22, 23]. This poor integration not only exacerbates fragmentation in health care delivery [14], but it perpetuates social injustice within human resources, as CHWs often lack opportunities to contribute to important decision-making processes [6, 20] and to get promoted within their roles [8, 23]. As their engagement within health systems becomes more commonplace, questions arise over their long-term position in health systems [14] and, more broadly, whether they can mitigate HIV through a means that does not exploit or overlook their important roles within HIV programming. Ethical and practical guidance therefore needs to become the cornerstone of CHW deployment. Prioritizing this approach can aid stakeholders in navigating the complex challenges arising in CHW programs.

Currently, however, much of the literature on this topic focuses on the efficacy and applicability of CHWs responding to specific health issues [11], or the potential health system challenges that can be alleviated by task shifting [14]. Little research has focused on the harms or ethical and practical issues [17, 18, 20] that may arise when engaging CHWs in health service provision. Research therefore risks being one-sided, as the metrics used to showcase CHW effectiveness may not be comprehensive. This paper seeks to shed light on this critical knowledge gap by examining ethical challenges that emerge when CHWs are deployed within HIV programs in low-income contexts. To better understand how these challenges have come into fruition, this paper overviews these challenges in the context of CHW programs in low- and middle-income countries (LMICs). Specifically, we first outline how CHWs historically emerged in HIV care delivery and the role they played in filling service delivery gaps existing in under-resourced health systems. A set of ethical principles is then introduced, which, if attended to, can better ensure that CHWs are deployed in a respectful and non-exploitative manner.

## Methods

To compile a list of principles that showcase which principles need to be upheld in HIV programs that involve CHWs, the authors first analyzed ethical challenges emerging in HIV service delivery. To do this, we searched the peer-reviewed literature using MEDLINE, Cochrane Database of Systematic Reviews, and Google Scholar databases using the following key terms: “Community Health Workers,” “HIV,” and “Ethics” It should be noted that several terms are currently used in peer-reviewed literature to describe CHWs. Some of these include lay health workers [10, 14], home-based carers [12], peer supporters [24], treatment supporters [10, 11], health promoters [25], village health workers [7, 9, 11], and lay counsellors [2, 12, 26]. For this paper, the central term used to conduct the literature search was CHWs, as since 2004, it has been widely referred to as being an overarching concept for lay workers who receive basic training and are employed in the health sector [12]. We examined six articles from reference lists to identify additional relevant articles that were missed via our search strategy. Articles included in the search were those published in year 2000 to 2017, were written in English, discussed CHWs and their usage in HIV care delivery, examined these challenges in LMICs, or discussed the concept of ethical principlism. In total, 465 articles were identified, 78 of which were included for in-depth review. These articles were reviewed with relevance to the following question: *What are the practical and/or ethical challenges that CHWs can encounter in their work?* To ensure we gained a nuanced understanding of these challenges, grey literature including WHO reports and reference lists of the identified studies were also examined.

As showcased in the literature, several ethical challenges arise when CHWs are used to combat the spread of HIV in LMICs. These issues are multifaceted and often interconnected. To better understand these complex challenges, data were analyzed from the perspective of ethical principles. As Holland argues [27], principlism can be a strategy to better understanding and dissecting ethical issues arising in health service provision. By identifying challenges, and the contributing factors that may lie behind them, we can more clearly propose strategies to help resolve them. The five ethical principles that will be expanded on in this paper, which have particular relevance to CHW and HIV programming include: respect for persons, justice, beneficence, proportionality and cultural humility. After closely reviewing existing principles used in medical and public health contexts [20, 28–31], these five principles best reflected the range of ethical challenges covered in this paper. Further justification for this decision is provided in the principlism section.

## Results

### History of CHWs in LMICs

By definition, CHWs are individuals without formal tertiary education who are selected by community members to receive basic training in order to provide one or more health care functions in their communities [10]. To understand the role of CHWs in low- and middle-income health systems, along with ethical challenges that may accompany their work, one must first examine how their usage came about. Widespread involvement of CHWs is not a new phenomenon [2, 4, 11, 12, 16]. The first CHWs can be traced back to rural China in the 1920s where community members received basic education to carry out health activities [32] such as recording births and deaths and providing vaccinations [13]. This concept gained further attention around the world in the 1960s when international stakeholders began viewing this model as a strategy to improving access to healthcare services in rural areas where trained physicians often failed to reach. This concept began slowly being introduced in communities in other LMICs [13].

In the 1970s, multilateral agencies such as the WHO began exploring ways to use task shifting as a formal strategy to strengthen health outcomes in resource-limited contexts [13]. This was most notably reflected in the 1978 Alma Ata declaration which envisioned CHWs as representing a pragmatic way of enhancing community involvement in health care delivery [7]. This perception continued into the early 1980s where CHWs were viewed as being a cornerstone of the primary health care movement [7, 9, 13]. However, by the late 1980s and early 1990s, enthusiasm for CHWs began to quickly diminish [7, 12, 13]. This perceived lack of usefulness led to decreased interest in CHW programs, which mainly arose due to lack of adequate planning, training, management, and funding being incorporated into programs [4, 11]. Challenges with scale-up were therefore becoming apparent [7, 13], as concerns arose over their long-term role within health systems [12]. These constraints were ultimately impacting the quality of care being delivered [33], as poor motivation and high attrition rates were becoming commonplace in CHW programs [34]. During this time, key challenges began to arise, which were often a result of poor health financing and inadequate supervision from other health worker cadres [35]. A debate thus began to surface over whether CHWs could indeed become mediators of health behaviour change or rather represented “narrow functionaries of the health system.” [12]

However, by the mid-1990s, a resurgence of CHW programs was observed. This was sparked by several factors, most notably, the rapid spread of the HIV epidemic [10–12] and growing interest in health care decentralization [10, 11]. More specifically, the HIV crisis caused many highly trained health workers to experience increased workloads, as they were expected to test people for HIV, place individuals living with HIV on a continuum of treatment services, and, by the mid-2000’s, roll-out ARTs while simultaneously tending to other duties [36]. These cumulative responsibilities began exacerbating rates of worker absenteeism in health systems [1, 37], which was compounded by other issues including health worker migration and concerns that providers may become infected with HIV themselves [38]. CHWs represented a cost-effective way of remedying some of these complex issues and were therefore increasingly deployed in HIV programs [2, 39].

More recently, CHWs were viewed as a way of addressing critical human resource shortages, which are often a result of health workers migrating from low- to high-income countries in search of better work opportunities [1, 2]. The growing demands for CHW programs have also reflected efforts to achieve the Millennium Development Goals (MDGs) [1] and now the Sustainable Development Goals (SDGs). Lately, governments have made efforts to better integrate CHWs within health systems. For example, this has recently taken place in Tanzania, Brazil, Venezuela, Pakistan and Ethiopia [35, 40, 41]. Integrating CHWs into national structures can strengthen program compatibility with local practices, improve referral systems and health worker relationships, and strengthen overall health service delivery [41]. Yet despite this renewed interest in promoting CHW programs, older challenges persist. Some of these include poor training, supervision [35, 42] and lack of clear goals and priority setting [35]. These challenges are mainly a result of CHW programs being introduced in rushed, unstructured, and top-down manners [12].

As this overview showcases, shifting health care tasks to CHWs in LMICs is not new, as it emerged decades ago [2, 4, 11, 12, 16]. However, renewed interest in CHW programs has been “more pragmatic than ideological.” [12] This is because CHWs have been largely perceived as a means to curbing health system challenges [7, 12] like the HIV crisis, rather than a strategy to providing employment to lower educated citizens, or increasing community engagement in the health care decision-making affecting their lives. Furthermore, as shown in the provision of HIV services, task-shifting is often used to address specific health issues through focused interventions. In other words, CHWs reflect an attempt to fill health system gaps that arise from programs becoming increasingly verticalized and privatized in their approach [10, 11, 43]. These factors have ultimately undermined the ability and responsibility for governments to respond to persistent health system challenges [14, 43], and within this, CHWs have been used as a means to filling this void. Distinct political and philosophical underpinnings therefore lie behind the promotion of CHWs, which may explain why their central roles within health systems have gone largely unnoticed [7, 12]. As more stakeholders become involved in the response to the HIV epidemic, it is imperative that individuals and institutions recognize the complex, heterogeneous practices of CHW programs [14, 22] to better ensure their essential roles within health systems are not exploited or overlooked.

### Health system challenges affecting HIV programs in LMICs

Poor health financing is compounded in many LMICs by a large shortage of health workers. For instance, in Zambia and Myanmar, health spending accounts for only 5.35% and 4.95% of their total GDP [44]. These figures are near the average health expenditure rate across all LMICs, which is 5.37%; however, high income countries invest on average 12.38% of their GDP into health spending [44]. Moreover, only 9.1 physicians and 56.8 physicians are available in Zambia and Myanmar for every 100,000 people [45]. These figures are minuscule compared to countries like Canada or the United States, which have ratios of 253.9 and 256.8 physicians per 100,000 people respectively [45]. In many LMICs, of the few health workers that are available, they are unevenly distributed. For example, in Tanzania, nearly twice as many health personnel work in urban than rural areas [37], yet approximately 67% of Tanzanian citizens reside in rural parts of the country [46].

HIV prevalence rates vary across LMICs and are highest in sub-Saharan Africa, and in particular, Southern Africa. For example, in 2016, HIV prevalence among people aged 15 to 49 in Swaziland, Botswana and South Africa was 27.2, 21.9 and 18.9%, respectively [47]. Meanwhile, Southeast Asian countries such as Thailand and Indonesia have an HIV prevalence of 1.1 and 0.4% among adults aged 15 to 49. HIV prevalence is 0.5% in Guatemala and 2.1% in Haiti [47]. The last decade has witnessed extensive efforts to increase access to HIV care and treatment services, with varying regional success. For example, in Tanzania and Haiti in 2015, only 53% of of people aged 15 and over living with HIV had access to ART [48]. This figure is below the global targets set for HIV treatment, such as the United Nation’s (UN’s) 90–90-90 goals. More specifically, these goals aim to ensure that by 2020, 90% of people living with HIV will know their status, 90% of people living with HIV will be accessing treatment, and 90% of people on treatment will be virally suppressed [49]. Since only 53% of individuals over age 15 who are living with HIV are currently accessing treatment in these countries, much progress still needs to be made to achieve the UN’s second ‘90’ target. CHWs are viewed as having an important role in remedying this gap.

Although CHWs play a vital role in HIV service delivery, task shifting has occurred rather informally in LMICs, as little evidence showcases which tasks have been delegated from one cadre to another. For instance, poor recordkeeping and undefined roles in South Africa have caused many CHWs to perform duties outside their portfolio and skillsets [11, 50]. This has made it difficult to track how many CHWs are deployed in under-resourced health systems and can lead to further challenges. For instance, in settings such as the Mkuranga District of Tanzania, many village residents have reported not knowing that CHWs are working in their respective communities [9]. These issues may be a result of task shifting occurring as an ad hoc coping strategy to addressing health worker shortages [2, 21] rather than a systematic policy strategy. Important challenges are therefore impeding CHW’s abilities to provide timely, high-quality HIV services to targeted populations in LMICs. Within contexts where proliferation of new cadres has occurred rather rapidly, close consideration needs to be given to identifying and resolving practical and ethical challenges that undergird their work. One tangible way of achieving this is by using a set of ethical principles, which will be expanded on in the following section.

### Principlism

As showcased, CHWs represent feasible strategy for promoting community outreach [3], which has been fundamental in ART scale up [5, 16]; however, implementation concerns still persist. Some of these concerns include maintaining high standards of safety and quality care [2, 5, 11, 16, 21], reducing CHW attrition [1, 18, 51], standardizing training and supervision [5, 7, 8, 10, 21, 26, 52, 53], improving access to basic supplies [1], aligning CHWs with broad health system strengthening [5, 8], and prioritizing fair compensation [1, 5–7, 16, 19, 52]. These challenges cut across several CHW programs and are fraught with ethical concerns. To better understand these recurring issues, while further ascertaining ways to mitigate them, a set of ethical principles can be examined.

Principlism is a normative ethical framework which is used to navigate practical decision-making in the delivery of health care services [54]. As Coughlin highlights [29], principles play a prominent role in moral reasoning and can help reveal ethical underpinnings that may form the backdrop to many health problems. By specifically referring to a set of principles, an individual can more clearly elucidate a health dilemma while identifying a strategy to potentially resolve it [27]. In this sense, making principlist observations in health care can aid key stakeholders with pragmatically navigating ethical challenges that emerge in research- and practice-based settings [54].

Although originally developed in bioethics as a way of navigating individual cases where patient rights and autonomies are being breached, principlism can also be applied in public health contexts [27, 29, 54]. Unlike medicine, which is more individualistic in nature, public health is foremost concerned with promoting the health of populations by balancing the needs and desires of individuals, communities, and governments [27]. Common principles used to negotiate public health interactions include justice, trustworthiness, and respect [20, 27]. In this sense, principlism can provide a coherent analysis of the ethical issues that undergird public health interventions, as it can help stakeholders to examine and eventually resolve public health dilemmas [27]. This method of analysis is particularly useful in this paper as, currently, there appears to be no consensus on a structured way of addressing the aforementioned ethical challenges that emerge in CHW programs in LMICs.

What ethical principles should then be applied to guide HIV service delivery in CHW interventions in low- and middle-income settings? Many principles used in medical ethics can still be applied to public health contexts. For instance, as noted above, inadequate training and compensation can place unfair burdens on CHWs; this closely ties to the justice principle, which is often raised in biomedical ethics contexts, such as the work by Beauchamp and Childress [28] and in the Belmont Report [55]. Similarly, ensuring individuals like CHWs are provided with clear information so they can make informed decisions and properly conduct their tasks relates to the Respect for Persons principle raised in the Belmont Report [55]. Ensuring that welfare gains are maximized and that CHWs do not encounter unnecessary health risks as a result of their work can be covered in the beneficence principle [28, 55]. We have therefore included each of these high-level principles into our framework.

Mid-level principles are also relevant when working with CHWs in under-resourced contexts. For example, while some of the above principles may imply that fair remuneration be prioritized, these efforts will inevitably require additional resources, which can be hard to come across in healthcare delivery programs in many LMICs. The principle of proportionality [31] is therefore important to consider, as it can help program implementers and policymakers recognize that non-financial incentives, such as educational rewards and recognition by peers, may need to be considered. Lastly, HIV programs in LMICs are often conducted in collaboration between international and national stakeholders. This presents great opportunities for resources to be pooled; however, there is potential for miscommunications to arise in these cross-cultural contexts. Therefore, the final principle included is the principle of cultural humility, which we have adapted from Stones’ analysis of CHW programs in the United States [20].

### Respect for persons

Respect for Persons recognizes that all people have worth and therefore deserve to be respected. It acknowledges that every person has basic rights, such as their right to exercise their own autonomy and to make decisions without undue interference from others [28, 55]. Moreover, it has close ties to the Formula of Humanity, as it recognizes that all people have value and therefore should not be exploited or used merely as a means to achieving an institutional ends [56].

According to this principle, it is crucial that CHWs be given adequate information so they can exercise their full autonomy and make informed decisions. This is particularly important, as CHWs can often be in vulnerable positions due to their low levels of education and status compared to other health personnel. Despite this, some CHWs have reported being recruited into interventions without being given adequate training or information on how to properly conduct their tasks [9]. To circumvent this dilemma, when recruiting CHWs, they should be provided with clear instructions on the full scope of their tasks. They should also be told upfront whether they will be remunerated for their work; moreover, they can be notified whether there are opportunities for career advancement. Each of these endeavours can enable CHWs to make more informed decisions when undertaking their work.

Furthermore, it is critical for CHWs to not be viewed merely as a means to addressing gaps existing in HIV care delivery. One way of doing this is to recognize the limitations of CHW work; otherwise, CHWs risk being undervalued and exploited in HIV programs. For instance, it is important to provide CHWs with workloads that are feasible and match their levels of expertise. Despite this, cases have been reported where CHWs feel overburdened due to their workloads being simply too much to manage [16, 51]. To circumvent this challenge, program implementers can interview CHWs throughout HIV programs to understand if their workloads are manageable and to identify if CHWs require further support. By taking these limitations into account, CHWs can become viewed not merely as a means to addressing gaps that doctors or nurses cannot fill, but rather as critical assistants who are providing important services in HIV programs.

### Justice

Justice is a principle that is shaped by the concept of respect but further adds to it [20]. Specifically, the justice principle argues that all people be treated fairly and given an opportunity to be heard [20, 30]. Justice therefore has important procedural implications, as it seeks to ensure that all stakeholders have equal opportunity to take part in procedural activities [20]. Likewise, it can have distributive impacts, as it strives to ensure the benefits and burdens of programs are distributed more fairly across all groups involved [28, 29].

In terms of procedural justice, while it is important for CHWs to receive ample guidance, supervision, and management from higher-educated health worker cadres, CHWs often receive few opportunities to provide feedback in HIV programs [6]. This highlights breaches in procedural justice, as institutional arrangements have perpetually excluded them from providing their input in HIV programs [20]. This issue can impact motivation and retention rates, as levels of dissatisfaction can arise from CHWs feeling as if they have few opportunities to provide feedback within HIV care delivery.

An effort to promote procedural justice was recently made in 2014 when CHWs were officially recognized as a professional health cadre in Tanzania when the Community Based Health Programme (CBHP) policy was approved. This policy tries to standardize and improve issues related to recruitment, training, employment, remuneration, supervision and performance assessment of CHWs [40]. Beforehand, CHWs had to carry out tasks with little support or resources from the public sector. By formally recognizing their role within the health system, there is greater potential for CHWs to receive new opportunities for negotiating health service terms [14] and experience more growth and promotion in their roles [8]. Although the integration process is impacted by several variables [6, 41], if done well, integrating CHWs into the health system represents a structured way to lessen fragmentation in HIV care delivery and address the larger crisis that is presented by a shortage of health workers [12]. This recent policy shift therefore can reflect an effort to promote procedural justice, as CHWs may experience greater opportunities to influence decision-making procedures as a result of this policy change.

According to distributive justice, efforts should be made to ensure that CHWs do not undergo unfair burdens while executing their tasks [20]. This challenge is ever more critical in LMICs, as many HIV programs are conducted between international and local stakeholders who may overlook context-specific burdens that CHWs may encounter from their work. For example, many CHWs report hopes of receiving financial rewards for their tasks yet are not fairly remunerated. In turn, CHWs may encounter unnecessary burdens, such as the need to rely on financial or material supports from their own family and/or community members so they can continue their work [19]. Similar challenges arise when CHWs are required to pay their own transport fees to perform a job or function for which they receive little or no compensation [19, 33, 50]. CHWs and local authorities should therefore be consulted so that burdens such as these can be identified, along with solutions to overcome them [33], such as the need for honoraria to be distributed.

Specific efforts can be made to promote distributive justice. Many, but not all, CHWs work as volunteers and therefore receive none or very little monetary compensation for their contributions [9, 20, 52]. Policymakers and program officers should thus pay close attention to the burden that lack of remuneration places on CHWs [19], and how setting a minimum standard of compensation can help to alleviate it. Other avenues can be explored, such as providing adequate equipment and resources [52] to avoid CHWs from encountering unfair burdens as a result of their work. Specifically, if CHWs have to travel long distances to carry out home-based counseling services, resources such as bicycles can be incorporated into program budget setting [19, 33]. Additional materials can be considered, such as umbrellas [33, 52], backpacks [33], and medications [52], for example. Adequate training and supervisory support can also be established to ensure that CHWs are delivering HIV services with enough preparation and guidance [8, 21, 52, 53]. Doing so can also promote greater collaboration and teamwork amongst health care providers, which is more reflective of task ‘sharing’ [57] than mere ‘shifting.’ Each of these endeavours reflect a steady effort to prioritize the justice principle. These efforts may also improve motivation, reduce attrition, and ultimately improve the quality of services that CHWs provide in HIV programs.

### Beneficence

According to the principle of beneficence, health and human welfare benefits should be promoted within the provision of health care services [28, 55]. Beneficence is often described in combination with non-maleficence, which aims to promote actions that mitigate harms or suffering of others [28]. According to this principle, public health interventions can be arranged so that they promote the well-being of individuals and communities, and minimize potential harms [29, 54]. The welfare gains may not only include improvements in health but social advantages such as community empowerment.

This principle has particular relevance to HIV programs. For example, in HIV service provision, CHWs may be placed in contexts where their own health may be at risk, such as conducting home-based HIV tests with inadequate protective equipment. Measures should therefore be taken to circumvent these issues. For instance, governments and institutions can ensure that CHWs are provided with proper equipment, such as latex gloves, when conducting HIV testing. This can allow CHWs to maintain and promote their health throughout their work.

Other circumstances may arise. For instance, peer educators (who are CHWs that are living with HIV themselves) risk being stigmatized from revealing their status when delivering HIV services [58]. CHWs should thus be provided with adequate training and support to ensure that peer educators do not endure unnecessary emotional harms or other forms of violence as a result of their work [20]. In public health contexts, it is equally important for CHWs to uphold this principle for wider social welfare gain. For instance, efforts can be made to protect community members from encountering undue social discrimination. It is therefore important for CHWs to be properly trained on ways to uphold and safeguard patient confidentiality throughout their work. Doing this will not only ensure that welfare is maximized and the beneficence principle is therefore being upheld, but it can also enable the respect for persons principle to be maintained. However, despite confidentiality being prioritized in HIV and CHW programs today, there are still circumstances where privacy is breached in HIV service delivery [59]. For example, instances have been recorded where CHWs have left personal data of HIV patients on their desks or in their cars [17]. It is therefore important that CHWs be reminded and provided with additional training so they can uphold patient privacy and confidentiality throughout their work.

### Proportionality

Proportionality is a mid-level principle that argues that public health benefits be weighed against moral considerations [30]. In other words, when making decisions, the principle of proportionality argues that all positive features be balanced against the negative consequences [31]. This principle is important in public health contexts, as it implies that individual benefits be considered within the context of wider social good [30]. In this sense, proportionality can enable decision-makers to evaluate a wide range of solutions and to choose the option(s) that are least infringing [31].

In HIV programs in LMICS, resource constraints need to be closely considered in decision-making processes. For instance, while it may be crucial for CHWs to be provided adequate compensation or supervision, program directors need to acknowledge that these activities will require more resources, which can be difficult to come by in LMICs [21]. While the justice principle argues that fair remuneration be prioritized, in contexts where fiscal constraints deter CHWs from receiving standardized salaries, alternative non-financial incentive strategies can be explored, such as providing CHWs with flexible hours, strong management, or educational rewards [7, 33]. Providing CHWs with training opportunities may also result in greater quality HIV services being provided to patients; this is a wider social good that may outweigh the extra time and costs required for this training. There are other non-financial rewards. For example, CHWs can experience an increased sense of leadership and community connectedness from their work; moreover, they can be provided with more clearly defined roles, and supervisors can deliberately recognize CHW contributions [41, 51].

The principle of proportionality also implies that CHWs be given a fair workload and level of responsibility in comparison to their skillsets. While it is important to acknowledge that different levels of staff will inevitably exist in HIV programs, ethical and practical challenges can emerge when CHW are assigned tasks that do not proportionately match their levels of expertise. According to this principle, it therefore becomes more apparent that CHWs be delegated tasks that match their skillsets so they can feasibly carry them out.

### Cultural humility

The principle of cultural humility emphasizes the need for stakeholders to be open to exchanging cultural knowledge and skills throughout healthcare delivery to ensure that greater collaboration and partnership-building is promoted [20]. This principle is particularly important in HIV programs in LMICs, as they are often carried out between individuals from various cultural backgrounds. For instance, HIV programs may involve local and international stakeholders representing NGOs, universities, government officials, and community members. Although transnational programs present an enormous opportunity for more resources to be pooled, if context-specific burdens are not considered, CHWs may disengage from their work [33]. Misunderstandings can also arise if international stakeholders lack appropriate cross-cultural skills. It is therefore important for program designers, implementers and other relevant stakeholders undergo cultural competency training [20]. This can better ensure that individuals are open to hearing and critically reflecting on cultural nuances that may affect HIV service delivery.

Another tangible way of promoting the cultural humility principle is allowing regular interviews or meetings to take place with CHWs in HIV programs, along with avenues to implement suggestions and recommendations. These interviews can shed light on cultural nuances that may be overlooked in service delivery. For instance, interviews with CHWs can reveal the need for transportation assistance to be provided, which may otherwise be overlooked and cause CHWs to become demotivated in their roles [33]. It is therefore important that an overall approach towards self-reflection and cultural humility be upheld in HIV service delivery. Within these arrangements, the main burden should arguably be placed on dominant institutions (rather than CHWs), since these stakeholders hold arguably more power in these relationships [20]. Thus, it is largely up to program designers and implementers to deliberately plan and incorporate opportunities for receiving feedback from CHWs within HIV programs.

### Limitations

As discussed, principlism represents a promising strategy to begin tackling ethical issues arising in public health contexts [27]. The five aforementioned principles offer a tangible way to navigate complex decision-making in HIV care delivery in LMICs. However, there are limitations to using principle-based approaches in public health settings. Principlism risks having a reductionist tendency [29, 54], as it reduces complex ethical issues into a concise list of principles. In doing so, some of the nuanced challenges that may undergird these issues risk being oversimplified [54]. Moreover, although principles serve as a guideline, they cannot always be strictly applied as there may be some contexts where they can be appropriately used and other instances where they cannot [29]. Additionally, principles are subjective, as they leave considerable space for judgment regarding what ethical issues ought to be prioritized [54]. The principles listed in this paper can therefore serve as a starting point to discussing and tackling public health ethical challenges [29] that emerge when CHWs are deployed in HIV programs. However, they do not represent a single, conclusive strategy to addressing the multifaceted issues discussed.

## Discussion

By acting as intermediaries between individuals and the formal health system, evidence shows that CHWs can improve access to basic health services [14]. CHWs represent a cadre that can help address systemic issues occurring within weak health systems, such as lack of community outreach [60] and health worker migration [2]. Although their roles in HIV care delivery are crucial, it is important to recognize that CHWs do not represent a “panacea” for weak health systems [7, 16, 43, 61]. Like any cadre, CHWs can experience limitations when it comes to their work, and if these issues are not recognized and addressed, ethical challenges can arise when it comes to their deployment.

To address these challenges, task shifting for HIV service delivery needs to occur with careful planning and consideration of the holistic and contextual factors that may impact HIV rates [39]. To do this, actions need to be taken to prioritize the principles of respect for persons, justice, beneficence, proportionality and cultural humility within CHW programs. More specifically, the contributions that CHWs bring should be better recognized [6] and respected. Ensuring that CHWs receive fair compensation [1, 5–7, 16, 19, 52] can represent one strategy to achieving this, as it shows that CHW contributions are valued as an important part of HIV service delivery. Improving working conditions is also critical [12]. For instance, CHWs should be provided with ongoing training and supervision to ensure they have adequate support and are not experiencing burn-out in their positions [16, 26]. Furthermore, closer attention should be given to considering any additional resources that CHWs may need, such as bicycles or other materials, to ensure they have the means necessary to carry out their tasks [19, 33].

Task-shifting must also be aligned with broad health system strengthening [5, 8]. For instance, recent progress was made to formalize CHWs as a cadre in Tanzania [40], with similar efforts made in Brazil, Venezuela, and Ethiopia [35, 41]. However, as Schneider et al. argue, formal integration of CHWs into national health systems is not a panacea to challenges associated with CHW programs, as this integration may limit possibilities for CHWs to participate in health work in other ways [12]. Further efforts can thus still be explored to better integrate their roles within health systems. For example, non-financial incentives can be incorporated into programs [33]. Another strategy is having non-governmental organizations (NGOs) and other institutions make concerted efforts to better coordinate their programs with the local government and municipalities [8, 35]. More specifically, these organizations can hire CHWs who are already volunteering in the health sector. In doing so, CHWs can experience increased capacities and opportunities for growth and promotion within their roles.

Additional efforts can be made to provide CHWs with opportunities to take part in decision-making processes. For example, starting from the planning stages, CHWs can be engaged and consulted in HIV programs to ensure their concerns and needs are being addressed [20]. This not only enables CHWs to have greater authority, but it can shed light on potential areas in need of improvement to in turn strengthen the standard of care being provided. In this sense, choosing to bring CHWs into decision-making processes can better ensure that HIV programs are being run in a manner that is feasible, culturally acceptable and empowering for all stakeholders involved.

The deployment of CHWs should also become better aligned with social policy to begin improving the quality of services in which they are providing [12]. Ethnographic work can shed light on ways to engage CHWs into policy-making decisions to better address social and economic inequities [36, 62]. Furthermore, steps can be taken to ensure that CHW remuneration and training is more standardized across HIV programs; this can create clearer career pathways for CHWs so they can experience long-term growth and employment [8, 12]. Lastly, given that the roles of CHWs are so essential in HIV service delivery, better coordination, political commitment, investment, and ownership needs to be provided on the government’s behalf [8]. Although recent efforts have been to formally recognize CHWs as a professional cadre in a handful of LMICs, close evaluation of these policy changes should take place so that stakeholders can identify where any shortcomings may exist which require further attention.

## Conclusions

HIV care delivery, like other public health matters, poses challenges causing trade-offs to be made between individuals, communities and governments [27]. The deployment of CHWs in HIV programs represents one example where such challenges can be brought into fruition. Although CHWs have emerged as a cadre to fill gaps existing in ART scale up and HIV care delivery [12], it is important to acknowledge that task shifting is not a single solution to the deeper, nuanced problems existing in resource-limited health systems [7, 16, 43, 61]. Stakeholders must therefore begin asking how CHWs can be engaged as effective agents for behaviour change *without* overstepping the boundaries of their responsibilities.

As we have showcased, several challenges arise in HIV programs that involve CHWs. Some of these include maintaining adequate training and supervision [5, 7, 8, 10, 21, 26, 52, 53], reducing attrition [1, 18, 51], upholding quality of care [2, 5, 11, 16, 21], and ensuring that CHWs are fairly remunerated for their work [1, 5–7, 16, 19, 52]. Principlism offers a pragmatic approach to assessing these challenges while further identifying strategies to alleviate them [29, 54]. The principles we have identified as being particularly relevant to the usage of CHWs in HIV service provision in low-and middle-income contexts include respect for persons, justice, beneficence, proportionality and cultural humility. By adapting these principles to CHW programs, stakeholders can more clearly reveal ways of ensuring that CHWs are being deployed through a means that do not overlook or exploit their important role in HIV care delivery. This is becoming ever more crucial as CHWs are clearly here for the future [20]. It is therefore imperative that concerted efforts be made to address the practical and ethical challenges that can undergird their work.

## Abbreviations

ART: Antiretroviral Therapy
cART: Combination Antiretroviral Therapy
CBHP: Community Based Health Programme
CHW: Community Health Worker
HIV: Human Immunodeficiency Virus
LMIC: Low- and Middle-Income Country
MDG: Millennium Development Goal
NGO: Non-Governmental Organization
SDG: Sustainable Development Goal
UN: United Nations
WHO: World Health Organization
